# Health-related quality of life measurement in pediatric clinical practice: An appraisal and precept for future research and application

**DOI:** 10.1186/1477-7525-3-34

**Published:** 2005-05-16

**Authors:** James W Varni, Tasha M Burwinkle, Mariella M Lane

**Affiliations:** 1 Department of Pediatrics, College of Medicine, Department of Landscape Architecture and Urban Planning, College of Architecture, Texas A&M University, 3137 TAMU, College Station, TX 77843-3137 USA; 2 Department of Anesthesiology, University of Washington, 1945 NE Pacific Street, Seattle, WA 98195 USA; 3 Department of Psychology, College of Liberal Arts, Texas A&M University, College Station, TX 77843 USA

**Keywords:** health-related quality of life, clinical practice, pediatrics, children, healthcare services

## Abstract

Health-related quality of life (HRQOL) measurement has emerged as an important health outcome in clinical trials, clinical practice improvement strategies, and healthcare services research and evaluation. HRQOL measures are also increasingly proposed for use in clinical practice settings to inform treatment decisions. In settings where HRQOL measures have been utilized with adults, physicians report such measures as useful, some physicians alter their treatment based on patient reports on such instruments, and patients themselves generally feel the instruments to be helpful. However, there is a dearth of studies evaluating the clinical utility of HRQOL measurement in pediatric clinical practice. This paper provides an updated review of the literature and proposes a precept governing the application of pediatric HRQOL measurement in pediatric clinical practice. Utilizing HRQOL measurement in pediatric healthcare settings can facilitate patient-physician communication, improve patient/parent satisfaction, identify hidden morbidities, and assist in clinical decision-making. Demonstrating the utility of pediatric HRQOL measurement in identifying children with the greatest needs, while simultaneously demonstrating the cost advantages of providing timely, targeted interventions to address those needs, may ultimately provide the driving force for incorporating HRQOL measurement in pediatric clinical practice.

## Review

The last decade has evidenced a dramatic increase in the development and utilization of health-related quality of life (HRQOL) measures in an effort to improve patient health and determine the value of healthcare services [1]. Most research efforts on HRQOL in medical decision-making thus far have focused on measuring patient-reported outcomes (PROs), healthcare utilization, and the incorporation of HRQOL assessments in clinical trials with the goal of identifying group discrimination between treatments. Unfortunately, a dearth of empirical peer-reviewed studies exists that have evaluated the clinical utility of HRQOL measurement in pediatric clinical practice.

The use of HRQOL measurement in clinical practice has focused primarily on adult patients. In settings where HRQOL instruments have been employed with outpatient adults, physicians report that such measures are useful, and that they use PROs to alter treatment. Patients generally perceive the instruments to be helpful in communicating their healthcare needs to their physicians [2,3]. Further, a number of position papers have suggested that the routine assessment of HRQOL in clinical practice may facilitate best practices [4,5], with the caveat that the routine use of HRQOL assessment should be considered within the overall framework of evidence-based practice [6].

This paper serves to provide an updated review of the literature and to build a rationale for the incorporation of HRQOL measurement into pediatric clinical practice by evaluating both conceptual arguments for its benefit and existing empirical evidence of outcomes for its use in clinical practice. Since a paucity of data exists on these issues in pediatric clinical care, the adult literature is reviewed and implications for pediatric settings are addressed. Logistical issues related to incorporating such assessment procedures into clinical settings are then examined, and recommendations are made for future research and practice.

## Rationale and evidence for the incorporation of HRQOL measurement in clinical practice

### Facilitation of patient-physician communication

One potential benefit of incorporating HRQOL assessment into clinical practice is the impact on patient-physician communication [7]. The adult literature supports the idea that both patients and physicians believe HRQOL feedback to physicians assists communication [3,8-11]. For example, patients are often willing to discuss HRQOL with their physicians even when they are not experiencing significant impairments [8,12]. Unfortunately, although patients desire to share HRQOL information, physicians are not likely to ask [8,12-14]. In fact, research evidence suggests that physician behaviors (e.g., neglecting to ask about HRQOL issues) can impede a patient's willingness to introduce HRQOL concerns, that physicians vary greatly in their ability to elicit important information from patients, and that patients vary in their ability or desire to articulate it [15,16]. Patients are, however, generally willing to complete a HRQOL questionnaire at outpatient visits [8,12]. In this way, HRQOL measures can be a catalyst for communication in that they can objectively identify areas of concern that can then be discussed at the point of healthcare service.

Unfortunately, there are no randomized controlled studies in the published literature that have examined the impact of pediatric patient self-report or parent proxy-report HRQOL measurement on patient-physician communication. In pediatric practice, communication can be a key issue, as young children may not have the language skills necessary to accurately communicate their symptoms or feelings verbally. For these children, child self-report HRQOL measures that are sensitive to children's language and cognitive development may indicate problem areas, even those unrelated to the patient's chief complaint, that need to be further explored. In this way, one benefit of standardized HRQOL measurement in pediatric practice is the communication of healthcare needs by the child who may not be able to otherwise accurately report symptoms or other problems with daily functioning.

### Improvement in patient satisfaction

The adult literature suggests that patient satisfaction with medical care has been related to physician discussion of psychosocial topics and active physician elicitation of patient opinion [17,18]. Street and colleagues [13] reported that physicians who asked about pain, health perceptions, vitality, and role limitations due to physical problems were evaluated more favorably than physicians who did not. Another study by Brody et al.[19] reported that patients of physicians who received mental health feedback reported more satisfaction with their physician than patients in a control group. In the pediatric literature, no research has examined patient satisfaction and HRQOL assessment in pediatric practice. While it is unclear whether children would respond similarly, parents of pediatric patients are likely to report satisfaction or dissatisfaction with medical care based on their perceptions of their child's HRQOL outcomes as a result of treatment.

### Identification of hidden morbidities in pediatric clinical care

The adult literature has indicated a disparity in physician and patient ratings of health status [20-23]. Physicians frequently underestimate or fail to detect psychosocial and functional disabilities in patients, and often even high levels of psychological distress are undetected and untreated [22,24-28]. Studies with adult patients, however, indicate that physicians who receive HRQOL data have significantly higher detection rates for psychological problems [29-32], and for functional and HRQOL impairments [10,33]. With this information a physician may be better able to make appropriate referrals and tailor interventions to the specific needs of his/her patients.

Research in pediatric healthcare has also demonstrated the continuing under-identification of psychosocial problems, termed the "new hidden morbidity" [34], in routine pediatric practice [35,36]. Given the marked under-detection of these problems by physicians, HRQOL measures can serve as standardized screening instruments for identifying physical and psychosocial health concerns from the perspectives of both the child and parent at the point of service that pediatricians might otherwise overlook [37]. However, there are no existing randomized controlled pediatric trials that address this issue. Nevertheless, it seems reasonable to assume that pediatricians are likely to be assisted by standardized HRQOL measurement in the identification of hidden morbidities that might not otherwise be detected.

### Impact on clinical decision-making

In addition to traditional morbidity and mortality outcomes, evidence-based clinical decision-making requires validated patient-reported outcomes, particularly for chronic health conditions [38]. The impact of a HRQOL measurement instrument on clinical decision-making can be tested under the working hypothesis that HRQOL measurement and feedback to physicians must occur *at the point of service *in order to improve health outcomes [37]. Clinical decision-making may also benefit from a serial screening approach, which can assist with tracking changes in HRQOL over time. This longitudinal monitoring of HRQOL can help physicians evaluate the effectiveness of treatment or monitor disease status and associated symptoms such as pain, fatigue, coughing, wheezing, and the like.

In the adult literature, it has been estimated that up to 52% of physicians make management decisions (e.g., medication changes, ordering lab tests) on the basis of patient-reported HRQOL [6,8,39]. One study found that functional status feedback provided new information for approximately 25% of patients, resulting in specific clinical management actions by physicians in approximately 40% of those patients [3]. HRQOL measurement may also assist physicians with the referral process, in that they may be more likely to refer patients to mental health specialists or other health professionals as a result of this information [8,31].

Although these studies with adult patients suggest that improving detection through the use of HRQOL measurement does affect clinical care in various ways, the data regarding changes to clinical practice, after receiving HRQOL feedback, is inconclusive. For example, some adult studies have reported a lack of statistically significant differences between groups of physicians who received HRQOL feedback versus those who did not with regard to changes in prescription of medications, ordering of tests, referrals, or resource utilization [2,6,10,39-41].

The available evidence suggests that simply providing primary care physicians with HRQOL screening information without specific resource and management suggestions may not be sufficient in changing healthcare provider behavior [2,6]. Some physicians even express concern that HRQOL instruments measure some problems they are not prepared to treat; that is, they question the extent to which they could intervene, practically and financially, when certain problems are detected beyond their time and resources to address adequately [42,43]. The failure to produce changes in treatment may also be related to physician's limited experience with HRQOL measures or difficulty in translating HRQOL data into specific interventions to improve functioning.

The clinical utility of HRQOL measurement in clinical practice is likely to be enhanced by the availability of measures that are easy to use and interpret, physician education regarding the purpose and utility of HRQOL measurement, and specific management guidelines for deficits in HRQOL [6,44,45]. In fact, some investigators have concluded that the provision of targeted recommendations in conjunction with HRQOL scores are needed to yield beneficial effects of HRQOL measurement information on outcomes of care [8,40,46,47]. Providing practitioners with easy to use linkage tools (e.g., to appropriate referrals, resources, and evidence-based interventions) may greatly improve the integration of HRQOL data into clinical practice. However, linking HRQOL data to appropriate treatment recommendations, with subsequent improvements in HRQOL outcomes, has not yet been demonstrated empirically in pediatrics.

Thus, while the above studies provide valuable information about the impact of HRQOL on clinical decision making in adult patients, virtually no pediatric studies exist. One study with the Pediatric Quality of Life Inventory™ (PedsQL™) Generic Core Scales and Rheumatology Module in pediatric rheumatology [48], however, found that for those patients for whom the pediatrician reported that HRQOL measurement at the point of service influenced her clinical decision-making, subsequent HRQOL outcomes demonstrated significant improvement in comparison to patients in which HRQOL assessment was not utilized [37]. Nevertheless, this single empirical study only highlights the need for further research.

### Improvement in patient outcomes over time

A major hypothesized benefit to incorporating HRQOL measurement into routine clinical practice is the potential for identifying symptoms and problems that may result in improved patient outcomes over time. The findings are nevertheless mixed, with some physician-feedback intervention studies in the adult literature demonstrating no significant differences in patient outcomes such as functional status or health status between intervention and control groups [2,40,44,48,49], while other studies has yielded some support for improvement in mental health (e.g., depression) [10,48] and role functioning [10] following feedback interventions.

Overall, the results from the adult literature suggest that routine implementation of standardized HRQOL screening may be a necessary but not sufficient condition for enhancing patients' HRQOL. For example, Rubenstein and colleagues [47] reported that providing physicians with patient functional status information in conjunction with management and community resource suggestions resulted in better mental health and social activity scores. Thus, incorporating specific resource management suggestions, such as appropriate referrals and tailored treatments, may enhance the potential potency of HRQOL measurement by providing physicians with variable options to identified problems.

In sum, the adult research on the integration of HRQOL measurement into clinical practice has demonstrated that the provision of HRQOL data to physicians is more likely to impact detection and diagnosis, but is generally not sufficient in changing physicians' management behaviors unless the HRQOL findings are linked to the available resources necessary to improve HRQOL outcomes. The general failure to produce changes in treatment may be related to physician's limited experience with such measures or the difficulty of healthcare professionals in translating HRQOL data into specific interventions to improve functioning. Finally, there may be significant barriers to the implementation of HRQOL measures into clinical practice, as discussed next.

## Barriers to incorporating standardized HRQOL measurement in clinical practice

Barriers such as perceived lack of time, money, and human resources needed to collect, analyze, and interpret HRQOL data, as well as the lack of ongoing computer support for storing and retrieving data, can greatly impede implementation of routine HRQOL measurement in clinical practice [6,50]. Thus, while HRQOL measurement logically appears to have potential utility in pediatric clinical practice, there are a number of perceived barriers to use that must be addressed, including that: (1) measures may be perceived as too long, impractical, and difficult to score for a clinic with multiple time constraints; (2) physicians may not be convinced that HRQOL is related to clinical signs and symptoms, which traditionally have been the focus of clinical intervention; (3) implementation of HRQOL measurement may be perceived too costly, requiring additional resources such as staff time and computer scoring systems; (4) implementation of HRQOL measurement is perceived as potentially interfering with clinic operation, and (5) the information provide by HRQOL instruments is perceived to be already available through conventional testing methods (e.g., semistructured questions about health and symptoms).

### Concerns about resources

Based on their semi-structured interviews of physicians concerning practice-based functional health assessment, McHorney and Bricker [42] found that physicians desired a point-of-service data collection process that did not interrupt the flow of care; perceived barriers included insufficient staff and computing resources, as well as the need for summary output to be able to be immediately implemented. A study in an outpatient oncology clinic addressed several of these issues, reporting that completion, scoring, and printing of patient-reported HRQOL data could be conducted during waiting room time [9]. Additionally, summaries provided to physicians did not lengthen average consultation time, and anecdotally, some physicians expressed the belief that the summary increased their efficiency [9]. Thus, intervention studies comparing duration of physician consultation have found that having access to HRQOL data does not necessarily increase the amount of time spent with patients [9,10].

The application of computer-assisted assessment technology to the measurement of patient self-report and parent proxy-report in pediatric clinical care may reduce a number of the perceived barriers associated with the administration, completion, and scoring of standardized HRQOL instruments, and consequently represents one method for potentially overcoming several barriers to the use of these measures in pediatric practice. Computer-assisted HRQOL measurement as a screening methodology may facilitate the identification of areas of concern at the point of service, eliminate a lengthy interview process, and assist with targeting interventions [51-54]. For instance, a one page electronically-generated report can provide a brief summary of symptoms and problem areas for the individual patient on a real time basis, which may influence clinical decision-making at the point of service. This summary report could also generate targeted referral suggestions to facilitate the clinical decision-making process and make suggestions for targeted evidence-based healthcare based on the HRQOL findings.

### Attitudinal barriers

One attitudinal barrier to the incorporation of HRQOL assessment in clinical care is clinicians' views of the relevance and value of this information in patient care. Physicians vary greatly in their degree of willingness to discuss HRQOL issues with patients, and this willingness impacts patients' behavior (e.g., patients do not discuss their HRQOL concerns with their physician unless prompted to do so) [12,16,55].

Measuring the HRQOL of children in clinical practice may also meet with some resistance by clinicians who feel that the completion of these measures represents too great a burden for children and their parents, particularly children with serious illnesses. Oftentimes, a more qualitative approach is sought, with the belief that such methods are less burdensome and intrusive than standardized quantitative methods [56]. While an understandable concern, similar concerns have been seen in the past in regards to pain measurement in children [57] and HRQOL measurement in children with newly diagnosed cancer [58]. These concerns were met with brief instruments (to reduce respondent burden), developed with focus groups and cognitive interviews (to hear the voices of children and their parents), and by careful attention to the methodological details involved in establishing the reliability and validity of these instruments [59]. Subsequent to the development, field-testing, and documentation of the measurement properties of these standardized quantitative instruments, clinical trials have been conducted targeting pain management and enhancing HRQOL through symptom reduction using these quantitative instruments as outcome measures. Thus, standardized HRQOL instruments have the potential to improve the lives of children by providing the systematic documentation of the efficacy of treatment interventions designed for symptom relief [60]. In palliative care, for instance, standardized measures have the potential to increase the accountability and the quality of the care provided by comparing healthcare institutions or practitioners, and utilizing that information to inform consumers and aid quality improvement efforts [61].

A related attitudinal barrier to use in pediatric clinical practice is the perception by some physicians that HRQOL measures are neither sufficiently associated with nor predictive of subtle changes in physiological parameters [51,62]. For example, small-to-medium correlation effect sizes were found between perceived HRQOL and Hb_a1c _in children and adolescents with Type 1 diabetes [63], a finding consistent with the broader literature across diseases [64]. However, as succinctly summarized by McHorney [64]: "QOL scores correlate modestly at best with clinical outcomes. This finding suggests that clinical and human function are relatively independent. It does not imply that one or the other is inherently superior or correct. They simply measure different things, and using both will likely yield more information than any set alone" (p III-58). In fact, the use of physiological parameters to assess disease severity may be problematic, as many health conditions lack physiological markers, and such markers do not often apply across disease categories [65]. Furthermore, a patients' HRQOL may change in the absence of a measured biological change, or vice versa [62]. From this perspective, then, the incorporation of HRQOL measures into clinical practice should not be viewed as proxies for physiological parameters, but rather as a means of providing a more comprehensive evaluation of patient functioning across multiple life domains, particularly when meaningful physiological parameters are not available. This can be the hallmark for providing pediatric comprehensive care; multidimensional assessment leading to targeted interventions based on patient perceived needs.

As medicine shifts to a more patient-centered paradigm [38], willingness to incorporate HRQOL measurement is likely to increase. Physician education can be used to target the aforementioned attitudinal barriers, but Golden [45] notes the inadequacy of continuing medical education in changing physician behaviors and underscores the need for physician leaders who will advocate for and model the use of HRQOL measurement in clinical practice.

Finally, positive experiences with HRQOL measurement may reduce the aforementioned attitudinal barriers. Physicians in intervention studies who have used HRQOL measures report positive attitudes toward HRQOL assessment, expressing beliefs that the HRQOL data were accurate, were useful in clinical practice, could improve patient health status and satisfaction with care, provided new patient information, and facilitated physician-patient communication [2,8-10,29,39,40,44,46]. These positive experiences are a necessary but not sufficient condition for change, as detailed later.

## Selection of HRQOL instruments for use in pediatric clinical practice

For HRQOL instruments to be utilized in pediatric clinical practice, they must demonstrate their utility in the clinic setting by addressing the following criteria: (1) they must be brief, yet maintain reliability and validity and provide novel, valuable information; (2) they should be well-designed both for ease of use by patients/parents and for quick, easy scoring and interpretation; and (3) they must be responsive to meaningful patient change. While a comprehensive review of specific measures and their measurement properties is beyond the scope of this paper, interested readers may find reviews by Eiser & Morse [67] and Matza [68] helpful. Below we provide a briefly summary of the relative merits of generic and disease-specific measures, child and parent reports, and the measurement properties to consider in selecting an appropriate HRQOL measure for pediatric clinical practice.

## Generic and disease-specific HRQOL measures in clinical practice

There are potential advantages to an integrated modular approach combining the relative merits of generic and disease-specific HRQOL instruments [59,69,70]. A generic HRQOL measurement instrument allows for screening in healthy populations, enables standardized comparisons across pediatric chronic health conditions, and facilitates benchmarking with healthy populations [71,72]. While providing useful benchmarking data for comparisons with healthy children and across different disease groups, generic HRQOL measures may not, however, be as responsive to changes in disease-specific symptoms [73], and typically do not measure specific disease symptoms and treatment side effects of relevance to particular disease groups. In contrast, disease-specific measures (e.g., for asthma, cancer, diabetes, and arthritis) may be more sensitive to specific clinical changes in patients with a particular illness than a generic measure and may be particularly informative for pediatric chronic disease management at the individual patient level. Nevertheless, disease-specific instruments are unable to provide comparisons across diseases, including benchmarking with healthy population norms. Thus, there is an emerging perspective that for pediatric chronic health conditions, both generic and disease-specific HRQOL measures should be administered so as to gain as a more comprehensive evaluation of the patient's HRQOL.

## Child self-report versus parent proxy-report in pediatric practice

Imperfect agreement between self and proxy report, termed cross-informant variance [74], has been consistently documented in the HRQOL assessment of children with chronic health conditions and healthy children [75]. The existence of cross-informant variance and the subjective nature of HRQOL indicate an essential need for reliable and valid child self-report instruments for the broadest age range possible. However, while self-report is considered the standard for measuring perceived HRQOL, there may be circumstances when the child is too young, or too ill or fatigued to complete a HRQOL instrument, and parent proxy-report may be needed in such cases. Further, it is typically parents' perceptions of their children's HRQOL that influences healthcare utilization [35,76]. Thus, an instrument should be selected that meets the need to measure the perspectives of both the child and parent since these perspectives may be independently related to healthcare utilization, risk factors, and quality of care.

## Measurement properties requisite for individual patient interpretation

Key instrument properties that are important when HRQOL measurement is employed at the individual patient level include high internal consistency reliability (e.g., alpha ~ .90) and documented responsiveness to change over time as a result of treatment [77]. The establishment of clinically meaningful cut-points to identify at-risk patient status, and the documentation of the magnitude of score changes representative of clinically meaningful change, are essential measurement properties when using HRQOL instruments in clinical decision-making.

## Summary

The dramatic increase in use of HRQOL measures to evaluate patient outcomes and treatment alternatives reflects an emerging shift in health care toward valuing the patient perspective. However, only a few studies, primarily with adults, have evaluated the utility of HRQOL measurement in clinical practice. This research has demonstrated some support for the benefits of HRQOL measurement in facilitating patient-physician communication, improving patient satisfaction, increasing detection of problems with functioning, informing and altering clinical management, and improving patient outcomes. However, consistency in results is generally lacking, and similar data are virtually nonexistent for pediatric patients. Further, while the provision of HRQOL measurement feedback to physicians is likely to impact detection and diagnosis, it is often insufficient to change physicians' management behaviors (and subsequently improve patient outcomes) unless HRQOL findings are linked to suggestions for specific interventions, including appropriate referral sources.

Routine use of standardized HRQOL instruments in pediatric clinical practice may be impeded by physician attitudinal barriers or concern that completion of these measures represents too great a burden. Further, physicians desire information about the effectiveness of practice-based functional health assessment and are reluctant to routinely use HRQOL measurement if evidence is lacking that it improves patient clinical outcomes. Intervention studies are needed to provide evidence-based demonstration supporting the setting aside of valuable resources needed for the integration of HRQOL measurement in pediatric clinical practice. Intervention studies that provide physicians with targeted recommendations linked to HRQOL data are especially needed.

## A precept for future research and practice

What is the overarching precept or principle that should guide future research in determining the utility of HRQOL measurement in pediatric clinical practice? We propose that while doing the right thing for children is always the foremost principle, the realities of today's healthcare industry mandates that any change to current standard practice demonstrate that the change either does not cost more to the organization, or even more ideally, saves the organization costs in the future.

While cost savings are rarely the sole driving force behind change in pediatric healthcare systems (the corollary to the above is typically true, i.e., cost savings should have no negative impact on care, and ideally improve clinical care through greater efficiencies and reduced variances in the provision of care), convincing senior management to implement a change that has upfront costs is challenging at best. Children's Hospitals and pediatric healthcare systems often find themselves struggling to survive during economic downturns. Without clear economic value, as perceived by senior management, or regulatory or legislative mandate, changes in healthcare systems are rarely endorsed.

Thus, we suggest that the overarching precept for determining the likely implementation of HRQOL measurement in pediatric clinical practice requires a win-win circumstance, that is, in doing the right thing for children, the organization actually saves healthcare cost in the future, or at the very least, finds the change "revenue neutral" (i.e., the change pays for itself somehow).

What are the conditions under which such a set of circumstances could be achieved? We believe that nuggets of evidence exist in the literature that portend the set of circumstances above, although only suggestive thus far. Specifically, prospective cohort studies that have demonstrated the utility of HRQOL measurement in predicting future healthcare utilization and costs suggest the necessary but not sufficient condition for the overarching precept above. For example, in the adult literature, lower asthma-specific HRQOL was associated with higher risk of asthma-related emergency department visits or hospitalizations and higher healthcare costs during the subsequent 12 months [78]. In pediatric patients, generic HRQOL (PedsQL™) prospectively accounted for significant variance in healthcare costs at 6, 12, and 24 months [79]. Adjusted regression models that included both HRQOL scores and chronic health condition status accounted for 10.1%, 14.4%, and 21.2% of the variance in healthcare costs at 6, 12, and 24 months. HRQOL and chronic health condition status together defined a 'high risk' group, constituting 8.7% of the sample and accounting for 37.4%, 59.2%, and 62% of healthcare costs at 6, 12, and 24 months. The high risk group's per member per month healthcare costs were, on average, 12 times that of other enrollees' at 24 months.

Pediatric risk prediction is of increasing importance for healthcare providers, purchasers, payers, and policy makers. Predicting resource utilization is key to managing defined populations in a prospective payment system and for proactively case-managing those at greatest risk of poor health. If providers knew in advance, for example, at health plan enrollment which children were most likely to become ill, health care resources could be proactively targeted to those children in order to minimize or prevent that morbidity and associated costs. Being able to demonstrate the utility of pediatric HRQOL measurement in identifying children with the greatest needs, while simultaneously demonstrating the cost advantages of providing timely, targeted interventions to address those needs, may ultimately provide the driving force for incorporating HRQOL measurement in pediatric clinical practice.

Thus, the findings above suggest that pediatric HRQOL data can be used to prospectively predict healthcare costs. When combined with chronic health condition status, HRQOL data can identify an at risk group of children as candidates for proactive care coordination. The win-win proposition entails doing the right thing for children when their needs are identified at the point of healthcare contact, with the potential for healthcare cost savings in the future for the healthcare industry. It seems like a precept worth testing in the service of children and society.

## List of abbreviations

HRQOL Health-Related Quality of Life

PedsQL™ Pediatric Quality of Life Inventory™

## Authors' contributions

All authors contributed to the conceptualization and drafting of the manuscript. All authors read and approved the final manuscript.
